# Lifetime Swimming Pool Attendance and Cancer Risk: Findings from the Multicase-Control Study in Spain (MCC-Spain)

**DOI:** 10.1021/acs.est.5c06488

**Published:** 2025-11-03

**Authors:** Carolina Donat-Vargas, Miquel Vallbona-Vistós, Gemma Castaño-Vinyals, Víctor Moreno, Nuria Aragonés, Elena Boldo, Antonio José Molina de la Torre, Inés Gómez-Acebo, Marcela Guevara, Ana Jiménez Zabala, Pilar Amiano, Ana Molina-Barceló, Guillermo Fernández-Tardón, Maria Dolores Chirlaque, Marina Pollán, Manolis Kogevinas, Cristina M. Villanueva

**Affiliations:** † 310844 ISGlobal, Doctor Aiguader 88, 08003 Barcelona, Spain; ‡ Polyphenol Research Group, Department of Nutrition, Food Sciences and Gastronomy, School of Pharmacy and Food Sciences, University of Barcelona, Campus Diagonal, Av. de Joan XXIII, 27-31, Distrito de Les Corts, 08028 Barcelona, Spain; ○ INSA-UB, Nutrition and Food Safety Research Institute, Carrer de Prat de la Riba, 171, 08921 Santa Coloma de Gramanet, Spain; § CIBER de Epidemiología y Salud Pública (CIBERESP), Instituto de Salud Carlos III, Av. Monforte de Lemos, 3-5, Pabellón 11, 28029 Madrid, Spain; ∥ Unit of Cardiovascular and Nutritional Epidemiology, Institute of Environmental Medicine, Karolinska Institutet, Nobels väg 13, 171 65 Solna, Stockholm, Sweden; ⊥ Universitat Pompeu Fabra (UPF), Doctor Aiguader 88, 08003 Barcelona, Spain; # IMIM (Hospital del Mar Medical Research Institute), Doctor Aiguader 88, 08003 Barcelona, Spain; ¶ University of Barcelona, Feixa Llarga s/n, 08907 L’Hospitalet de Llobregat, Barcelona, Spain; ⊙ Catalan Institute of Oncology, Bellvitge Biomedical Research Institute (IDIBELL), Gran Via km 2.7, 08907 L’ Hospitalet de Llobregat, Barcelona, Spain; ◑ Cancer Registration & Surveillance Unit, Public Health Division, Department of Health of Madrid, C/López de Hoyos 35, 28002 Madrid, Spain; ⌽ 38176 Carlos III Institute of Health, Monforte de Lemos 5, 28029 Madrid, Spain; ◊ Research Group in Gene−Environment−Health Interactions (GIIGAS), Institute of Biomedicine (IBIOMED), University of Leon, Campus de Vegazana s/n, 24071 León, Spain; ⬢ Preventive Medicine Group, University of Cantabria, 39011 Santander, Spain; ◆ IDIVAL-Valdecilla Health Research Institute, Avenida Cardenal Herrera Oria s/n, 39011 Santander, Spain; ⬠ Instituto de Salud Pública y Laboral de Navarra, Leyre 15, 31003 Pamplona, Navarra, Spain; □ Navarra Institute for Health Research (IdiSNA), C. de Irunlarrea, 3, 31008 Pamplona, Navarra, Spain; ◨ Public Health Division of Gipuzkoa, Biogipuzkoa Research Institute, Av. Navarra 4, 20013 San Sebastian, Spain; ⬓ Cancer and Public Health Area, Foundation for the Promotion of Health and Biomedical Research-Public Health Research (FISABIO), Avda. de Catalunya 21, 46020 Valencia, Spain; ⬔ Health Research Institute of Asturias (ISPA), Av. Hospital Universitario s/n, 33011 Oviedo, Asturias, Spain; ☆ Department of Epidemiology, Murcia Health Council, IMIB-Arrixaca, Ronda de Levante 11, 300008 Murcia, Spain; ◮ Department of Health and Social Sciences, Universidad de Murcia, Av. Teniente Flomesta 5, 30003 Murcia, Spain

**Keywords:** Disinfection byproducts, swimming pool, breast cancer, colorectal cancer, prostate cancer, observational study, case-control study, environmental epidemiology

## Abstract

Swimming in pools involves inhalation and skin absorption of potential carcinogenic disinfection byproducts (DBPs) as well as physical activity, which is protective for some cancer sites. We evaluated the association between lifetime pool attendance and the risk of breast, colorectal, and prostate cancer in a multicase-control study that recruited 4,941 hospital-based cancer cases (1,724 breast, 2,111 colorectal, 1,106 prostate) and 4,039 population-based controls in Spain (2008–2013). Lifetime swimming pool attendance in summer (as a surrogate of outdoor pools) and the rest of the year (as a surrogate of indoor pools), socio-demographics, and lifestyle were ascertained in face-to-face interviews. Cancer risk associated with pool attendance markers was estimated using linear mixed-effect models, adjusting for covariates with recruitment area as a random effect. Participants reporting lifetime pool attendance compared to those who did not showed lower odds of breast and colorectal cancer (approximately 5% lower risk). Swimming more than 10 times/month did not increase the protective association. For breast and colorectal cancer, only pool attendance outside the summer months was associated with a lower risk, whereas it was associated with increased prostate cancer risk. Findings suggest that lifetime swimming in pools may reduce breast cancer and colorectal cancer risk despite DBP exposure. These novel findings require replication.

## Graphical abstract

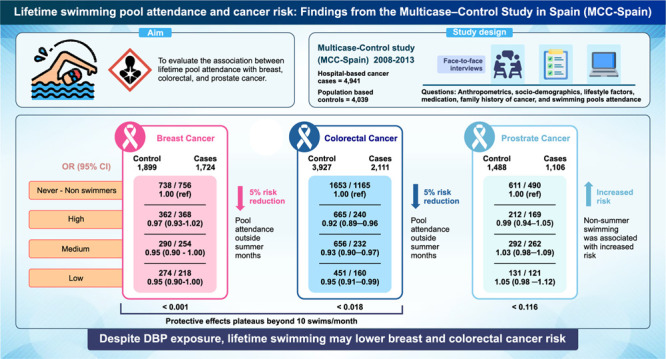

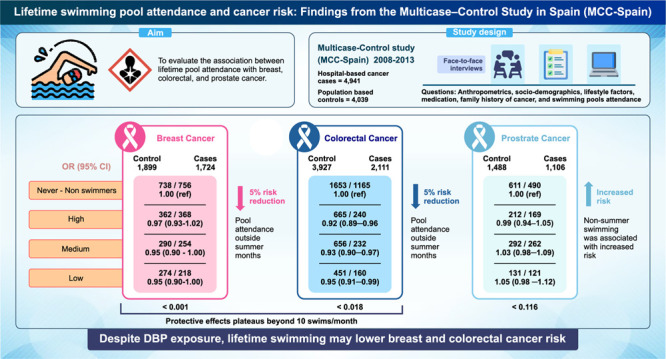

## Introduction

The use of disinfectants in swimming pools is essential to prevent waterborne infections.^1^ However, the unintended formation of disinfection byproducts (DBPs) through reactions between disinfectants and organic matter from swimmers (e.g., sweat, skin cells, urine, cosmetics, and other personal care products)^2,3^ is of health concern.^4,5^ Several DBPs have been shown to be genotoxic *in vitro* and carcinogenic in animal experiments,^6,7^ and the WHO International Agency for Research on Cancer (IARC) has classified some DBPs as possible human carcinogens.^8,9^

DBP exposure pathways during swimming, primarily through dermal absorption and inhalation, result in higher blood levels and longer persistence compared to oral exposure.^10^ We have reported uptake of trihalomethanes and haloacetic acids among swimmers using measurements in biological samples,^11^ which has been linked to increased genotoxicity,^2,12^ change in serum immune markers,^13^ blood transcriptional and microRNM responses,^14^ and metabolome changes^15^ after short-term exposure.^2^ Correlations between the concentration of various DBP classes and mutagenic potency in water have been found in chlorinated and brominated swimming pools and spas.^4,12,16^ However, there is limited evidence of potential cancer risk associated with long-term swimming pool attendance.

Breast, colorectal, and prostate cancers rank among the most prevalent malignancies worldwide.^17,18^ A number of lifestyle risk factors have been well-established such as diet for colorectal cancer^19^ and reproductive/hormonal factors for breast cancer,^19^ among others. Part of the burden of disease cannot be explained solely by these established risk factors and environmental exposures have been suggested as additional contributors.^19^ These cancer sites offer significant prevention potential through lifestyle modifications, and physical activity stands out as a key intervention accessible to large populations for cancer prevention.^20–23^

Swimming is a form of physical activity with low impact, making it suitable for individuals of all ages and fitness.^24^ Numerous studies have demonstrated the health benefits of swimming, including improvements in cardiovascular performance, muscle strength, and overall well-being.^25–27^ Research in mouse models suggests that swimming may have potential antitumor effects in colorectal cancer, possibly by inhibiting angiogenesis through suppression of the HIF-1α/VEGFA pathway.^28^ However, the environment in which swimming typically occurs–chlorinated and/or brominated swimming pools involving DBP exposure–introduces a potential health concern that warrants careful consideration.^29,30^

There is a need to clarify whether the potential cancer risk associated with exposure to DBPs in swimming pools might counteract the well-established benefits of physical activity during swimming. Therefore, we aimed to investigate the association between lifetime swimming pool attendance and the risk of breast, colorectal, and prostate cancers in a multicase-control study in Spain, including approximately 5,000 cancer cases and 4,000 controls.

## Materials and Methods

### Study Design and Population

The present study is part of the multicenter Multicase-Control study in Spain (MCC-Spain). Cancer cases were identified in hospitals from different Spanish regions through active searches in regular visits to hospital departments (i.e., gastroenterology, oncology, general surgery, radiotherapy, and pathology), and participants were interviewed as soon as possible after diagnosis (median 58 days) in 2008–2013. Minimal losses occurred from cases dying before being contacted (0.5% of potentially eligible cases). Controls were population-based and frequency-matched to cases by sex, age (±5 years), and region, identified from the lists of randomly selected family practitioners in primary health centers in the catchment area of participating hospitals.^31^ Inclusion criteria included age (20 to 85 years old), ability to understand and answer the questionnaire, and living in the study area for at least 6 months. The frequency matching was done separately for each study area, considering the age and sex distributions of the total number of cases recruited. The number of controls in the present analysis is larger than that in cases because of matching across different cancer sites. Response rates, calculated as the proportion of subjects interviewed out of all potential subjects (including those who refused), varied by region and cancer type. Average response rates were 71% for breast cancer, 68% for colorectal cancer, 72% for prostate cancer cases, and 53% for controls.

The protocol of MCC-Spain was approved by the ethics committees of the participating institutions. Information about ethics and the availability of data are offered at http://www.mccspain.org. Participants signed an informed consent form prior to enrollment. In addition, the database was registered with the Spanish Agency for Data Protection (no. 2102672171).

### Data Collection

Face-to-face interviews were conducted by trained personnel using computer assisted questionnaires to collect information from the study participants. Questions included anthropometrics (self-reported), socio-demographics, lifestyle factors, medication, family history of cancer, and swimming pool attendance, including ever attendance (arbitrarily defined as attending at least 10 times over a lifetime), average adult frequency, duration, and type of pool (indoor or outdoor), separately by summer and the rest of the year. The full questionnaire can be found online.^32^ Average diet corresponding to the year before the interview was collected through a self-administered semiquantitative food frequency questionnaire previously validated in Spain,^33^ including 140 food items. Interview reliability was assessed by the interviewer and recorded in the final section of the questionnaire.

### Outcome Definition

Cases were histologically confirmed incident cancer patients and included all malignant breast cancer [International Classification of Diseases (10th Revision); ICD-10: C50] and frequent *in situ* breast cancer (ICD-10: D05.1, D05.7). Incident colorectal cancer included ICD-10: C18, C19, C20, D01.0, D01.1, and D01.2 and prostate cancer, ICD-10: C61 and D07.5. The Gleason score for prostate cancer was collected from the pathological records. Two prostate cancer grading categories were constructed: low-medium grade (Gleason score <8) and high-grade/aggressive (Gleason score ≥8).^34,35^

### Swimming Pool Attendance

We evaluated lifetime swimming pool attendance by using multiple approaches. First, we considered it as a binary variable (ever vs never), defining “ever” as more than 10 lifetime visits. We categorized the average frequency (times/month) of pool use throughout life into tertiles based on the distribution among controls. Given that approximately half of the participants were nonswimmers, we created four categories: never (nonswimmers), low, medium, and high attendance. We also analyzed separately summer and nonsummer attendance patterns given different expected behaviors and pool characteristics (e.g., outdoor vs indoor).

### Covariates

Age was calculated based on birth and interview dates. Body mass index (BMI, kg/m^2^) was calculated based on weight and height 1 year before the interview. Smoking status was defined as having smoked at least one cigarette/day for ≥6 months in life, and former smokers were smokers who quit smoking ≥1 year before the interview. Physical activity was ascertained through open questions on any type of physical activity practiced in life, years, and frequency (hours/week) to calculate metabolic equivalents (METs) from age 16 to 2 years before the interview. In addition, we considered sex, education (less than primary school, primary school, secondary school, university), first degree family history of cancer, and use of nonsteroidal anti-inflammatory drugs. Among women, we considered oral contraceptive use, menopausal status, and hormone replacement therapy. The Gleason score was available for prostate cancer cases.

### Statistical Analysis

The initial study sample comprised 9,054 participants (1,738 breast cancer cases, 2,140 colorectal cancer cases, 1,112 prostate cancer cases, and 4,064 controls). We applied two exclusion criteria sequentially. First, we removed 35 subjects due to interviews deemed unreliable by trained interviewers, reducing the sample to *N* = 9,019. Subsequently, we excluded 39 participants due to incomplete swimming pool attendance data. The final sample for analysis consisted of 8,980 participants (1,724 breast cancer cases, 2,111 colorectal cancer cases, 1,106 prostate cancer cases, and 4,039 controls) (Figure S1).

We used mixed models with recruitment area as a random effect to estimate odds ratios (ORs) of cancer and 95% confidence intervals (CIs). To test exposure-response linear trends (*P* value for trend), exposure was treated as a continuous variable in the model by using the median concentration of categories. General additive models (GAMs) were used to display the exposure-response relationships on continuous variables (times per month) as a smoothed spline with three degrees of freedom.

We applied a three-tiered adjustment strategy to progressively control for potential confounders. (1) Baseline model: adjusted for matching variables and fundamental demographic factors (age, sex, and education) that are strongly associated with both swimming pool attendance and cancer risk. (2) Extended model: same as model 1) plus established cancer risk factors including family history of cancer (yes/no), smoking status (never, former, current), energy intake (kcal/day), red and processed meat intake (g/day), and BMI (kg/m^2^) to control for lifestyle and genetic predisposition. (3) Full model: additionally, incorporated physical activity (0, <8, 8–18, >18 METs·hour/week) to distinguish the effects of swimming from general physical activity benefits. Cancer-specific adjustments were also made based on established epidemiological evidence and biological plausibility; breast cancer models were further adjusted for oral contraceptive use (ever/never) and menopausal status and treatment (premenopausal, postmenopausal-never treated, postmenopausal-ever treated). Colorectal cancer models were further adjusted for sex and nonsteroidal anti-inflammatory drug use (ever/never). Additionally, we conducted stratified analyses by sex, physical activity level (<8 vs ≥8 METs·h/week), and menopausal status (pre- vs postmenopausal) for breast cancer, and tumor grade (low to medium-Gleason score <8 vs high-Gleason score ≥8) for prostate cancer. Interaction *p*-values were estimated based on the multiplicative term between ever vs never swimming pool attendance and the stratifying covariates.

Missing data for the following variables were addressed using multiple imputation:^36^ smoking (*N* = 42), BMI (*N* = 449), red and processed meat and energy intake (*N* = 1043), oral contraceptive use (*N* = 8), and menopausal status and treatment (*N* = 20). Multiple imputation uses information on all the variables included to replace missing values and generate different data sets with slightly different imputed values, allowing one to consider uncertainty. Analyses are done in all generated data sets, and results are pooled following Rubin’s rules.^1,2,36^ We performed multiple imputations by chained equations including all variables that are part of the analysis as well as additional variables that were related to the variables with missing values. Included variables were case/control status, age, sex, area, educational level, smoking status, BMI, red and processed meat intake, fruit and vegetable intake, fiber intake, vitamin C intake, vitamin E intake, alcohol intake, energy intake, ever nonsteroidal anti-inflammatory drug use, physical activity, menopausal status and treatment, ever oral contraceptive use, family history of cancer, ever attending the swimming pool, and swimming pool attendance frequency. We generated 15 different data sets using Predictive Mean Matching and polytomous logistic regression for continuous and categorical variables, respectively. The mentioned mixed models and GAMs were applied to the different data sets, and their results were pooled to obtain the final results.

Statistical analyses were conducted using R software (version 4.3.0). Mixed-effects models were fitted using the lme4 package (version 1.1.33); GAMs were fitted using the mgcv package (version 1.8.42), and multiple imputation was performed using the mice package (version 3.15.0).

## Results

There were no substantial differences between the cases and controls, except for a few notable distinctions. In the colorectal cancer sample, controls had a higher educational level compared to cases, while these differences were less pronounced in prostate cancer and nonexistent in breast cancer. Differences in the family history of cancer between cases and controls were observed. Lastly, the percentage of lifetime pool attendance was slightly lower among cases than controls, with more pronounced differences in colorectal cancer (Table 1).

**Table 1:** Characteristics of the Study Population^a^

	Breast cancer		Colorectal cancer		Prostate cancer	
Characteristics	Controls	Cases	Controls	Cases	Controls	Cases
*N*	1,899	1,724	3,927	2,111	1,488	1,106
Age (years), mean (SD)	59 (±13)	56 (±13)	63 (±12)	67 (±11)	67 (±9)	66 (±7)
Females, *n* (%)			1,923 (49%)	764 (36.2%)		
Males, *n* (%)			2,004 (51%)	1,347 (63.8%)		
Area, *n* (%)						
Madrid	365 (19.2%)	341 (19.8%)	726 (18.5%)	231 (10.9%)	334 (22.4%)	315 (28.5%)
Barcelona	392 (20.6%)	291 (16.9%)	1028 (26.2%)	690 (32.7%)	594 (39.9%)	403 (36.4%)
Navarra	179 (9.4%)	224 (13%)	263 (6.7%)	125 (5.9%)		
Guipuzcoa	255 (13.4%)	221 (12.8%)	352 (9%)	115 (5.4%)		
León	202 (10.6%)	223 (12.9%)	432 (11%)	379 (18%)		
Asturias	121 (6.4%)	68 (3.9%)	227 (5.8%)	75 (3.6%)	95 (6.4%)	16 (1.4%)
Murcia			42 (1.1%)	34 (1.6%)		
Huelva	78 (4.1%)	108 (6.3%)	174 (4.4%)	70 (3.3%)	94 (6.3%)	52 (4.7%)
Cantabria	183 (9.6%)	141 (8.2%)	349 (8.9%)	149 (7.1%)	175 (11.8%)	173 (15.6%)
Valencia	67 (3.5%)	61 (3.5%)	148 (3.8%)	81 (3.8%)	78 (5.2%)	84 (7.6%)
Granada			186 (4.7%)	162 (7.7%)	118 (7.9%)	63 (5.7%)
Girona	57 (3%)	46 (2.7%)				
Educational level, *n* (%)						
Lower than primary school	327 (17.2%)	268 (15.5%)	732 (18.6%)	678 (32.1%)	287 (19.3%)	260 (23.5%)
Primary school	583 (30.7%)	558 (32.4%)	1270 (32.3%)	796 (37.7%)	485 (32.6%)	436 (39.4%)
Secondary school	586 (30.9%)	567 (32.9%)	1116 (28.4%)	419 (19.8%)	404 (27.2%)	240 (21.7%)
University	403 (21.2%)	331 (19.2%)	809 (20.6%)	218 (10.3%)	312 (21%)	170 (15.4%)
Menopausal status and treatment, *n* (%)						
Premenopausal	547 (29%)	608 (35.4%)				
Postmenopausal-never treated	991 (52.5%)	876 (51.1%)				
Postmenopausal-ever treated	349 (18.5%)	232 (13.5%)				
Family history of cancer, *n* (%)	382 (20.1%)	498 (28.9%)	811 (20.7%)	597 (28.3%)	291 (19.6%)	314 (28.5%)
Body mass index (kg/m^2^), mean (SD)	25.8 (4.8)	26.1 (4.8)	26.7 (4.3)	26.5 (4.2)	27.5 (3.7)	27.4 (3.5)
Smoking, *n* (%)						
Never	1,140 (60.1%)	964 (56.3%)	1,740 (44.5%)	868 (41.3%)	397 (26.7%)	327 (29.7%)
Former	366 (19.3%)	336 (29.6%)	1,336 (34.1%)	839 (40.0%)	751 (50.6%)	527 (47.9%)
Current smoker	390 (21.6%)	413 (24.1%)	838 (21.4%)	392 (18.7%)	337 (22.7%)	247 (22.4%)
Physical activity, *n* (%)						
0 METs·hour/week	742 (39.1%)	757 (43.9%)	1,513 (38.5%)	954 (45.2%)	584 (39.2%)	451 (40.8%)
<8 METs·hour/week	358 (18.9%)	292 (16.9%)	597 (15.2%)	255 (12.1%)	195 (13.1%)	145 (13.1%)
8–18 METs·hour/week	241 (12.7%)	203 (11.8%)	468 (11.9%)	198 (9.4%)	170 (11.4%)	130 (11.8%)
>18 METs·hour/week	558 (29.4%)	472 (27.4%)	1349 (34.4%)	704 (33.3%)	539 (36.2%)	380 (34.4%)
Ever nonsteroidal anti-inflammatory drug use, *n* (%)	968 (51%)	779 (45.2%)	1791 (45.6%)	761 (36%)	656 (44.1%)	417 (37.7%)
Ever oral contraceptive use, *n* (%)	847 (44.5%)	768 (44.7%)				
Energy intake (kcal/day), mean (SD)	1,767 (±574)	1,878 (±662)	1,902 (±643)	2,018 (±712)	2,025 (±709)	2,079 (±695)
Red and processed meat intake (g/day), mean (SD)	79 (±52)	89 (±57)	94 (±61)	113 (±77)	109 (±63)	115 (±73)
Ever swimming pool attendance, *n* (%)[Table-fn t1fn5]	1,161 (61.1%)	968 (56.1%)	2274 (57.9%)	946 (44.8%)	877 (58.9%)	616 (55.7%)

a Number (%) of breast, colorectal, and prostate cancer cases and respective controls from the MCC-Spain. *n* (%) is presented for categorical variables and the mean (standard deviation, SD), for continuous variables. Percentages may not total 100 due to rounding. Total cases and controls for menopausal status/treatment and smoking do not match due to missing data: 20 missing for menopausal status/treatment; 42 missing for smoking. MET: Metabolic equivalents of task.

b Attended swimming pools at least 10 times in life.

Figure 1 and Table S1 show that participants who reported lifetime pool attendance, compared to those who did not, showed a slightly lower odds ratio of breast and colorectal cancer (approximately a 5% risk reduction) but not prostate cancer. The GAM (Figure 2) for both breast and colorectal cancer suggests an inverse association that appears linear until approximately 10 times per month (i.e., 2–3 times per week) mean attendance. Beyond this, higher attendance frequency is no longer associated with lower risk, and in the case of colorectal cancer, it may even increase risk. Table 2 displays the associations between cancer risk and categories of mean lifetime swimming pool attendance frequency (in times/month). Fully adjusted ORs (95% CI) for breast cancer comparing low (>0–3.2), intermediate (3.3–7.6), and high (>7.6) frequency vs never were 0.97 (0.93–1.02), 0.95 (0.90–1.00), and 0.95 (0.90–1.00). ORs (95% CI) for colorectal cancer comparing low (>0–2.1), intermediate (2.2–7.6), and high (>7.7) frequency vs never were 0.92 (0.89–0.96), 0.93 (0.90–0.97), and 0.95 (0.91–0.99). OR for prostate cancer comparing low (>0–2.0), intermediate (2.1–7.6), and high (>7.6) frequency vs never were 0.99 (0.94–1.05), 1.03 (0.98–1.09), and 1.05 (0.98–1.12).

**Figure 1:**
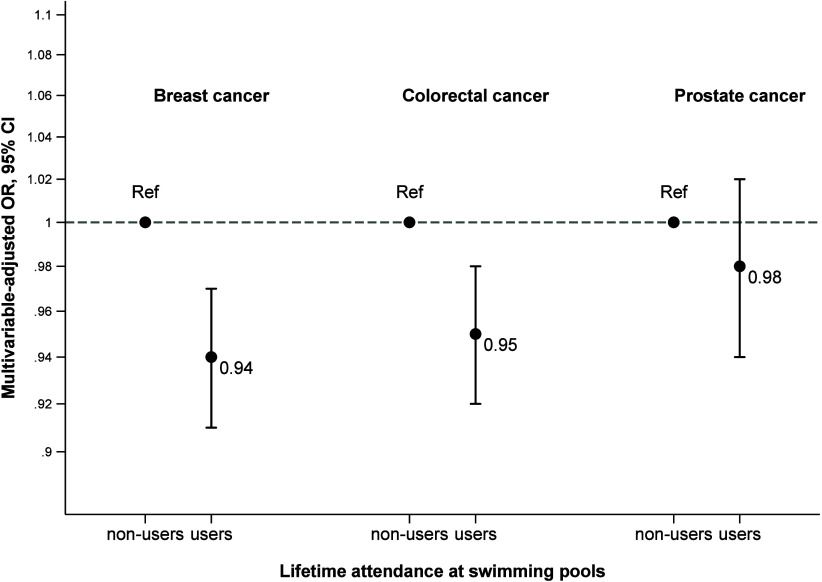
Association of lifetime pool users vs nonusers with breast, colorectal, and prostate cancers. Odds ratios (OR) and 95% confidence intervals (CI) were calculated using mixed models with residential area as random effect and were adjusted for age, sex, educational level, family history of cancer, smoking, energy intake, red and processed meat intake, body mass index, and physical activity. Breast cancer additionally adjusted for ever oral contraceptive use and menopausal status and treatment. Colorectal cancer additionally adjusted for ever nonsteroidal anti-inflammatory drug consumption. The *Y*-axis is in logarithmic scale.

**Figure 2:**
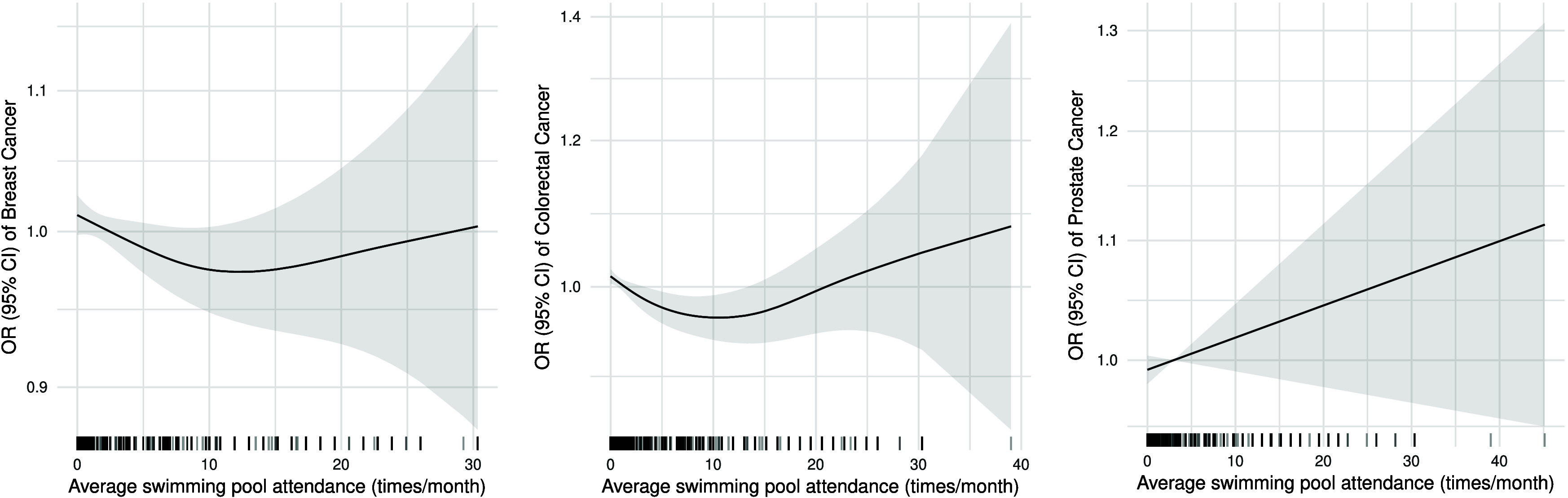
Generalized additive model plots for the association of average lifetime pool attendance with breast, colorectal, and prostate cancers. Odds ratios (ORs) and 95% confidence intervals (CIs). The black lines at the bottom of each panel represent the distribution of observed frequencies for average swimming pool attendance. The *Y*-axis is in logarithmic scale.

**Table 2:** Association between Breast, Colorectal, and Prostate Cancer with Average Lifetime Pool Attendance (Low, Medium, High vs Never)^a^

	Breast cancer^c^		Colorectal cancer[Table-fn t2fn4]		Prostate cancer	
Lifetime swimming pool attendance	Co/Ca	OR (95% CI)	Co/Ca	OR (95% CI)	Co/Ca	OR (95% CI)
Overall						
Never (nonswimmers)	738/756	1.00 (ref)	1653/1165	1.00 (ref)	611/490	1.00 (ref)
Low	362/368	0.97 (0.93–1.02)	665/240	0.92 (0.89–0.96)	212/169	0.99 (0.94–1.05)
Medium	290/254	0.95 (0.90–1.00)	656/232	0.93 (0.90–0.97)	292/262	1.03 (0.98–1.09)
High	274/218	0.95 (0.90–1.00)	451/160	0.95 (0.91–0.99)	131/121	1.05 (0.98–1.12)
*P* trend		<0.001		0.018		0.116
Summer						
Never (nonswimmers)	760/777	1.00 (ref)	1698/1184	1.00 (ref)	621/502	1.00 (ref)
Low	382/378	1.05 (0.97–1.14)	511/177	1.02 (0.96–1.09)	218/177	0.96 (0.87–1.07)
Medium	147/126	1.01 (0.92–1.12)	561/202	1.05 (0.99–1.12)	175/180	1.04 (0.93–1.16)
High	237/205	1.02 (0.93–1.11)	454/175	1.03 (0.97–1.10)	195/160	0.96 (0.86–1.07)
*P* trend		0.137		0.542		0.007
Nonsummer						
Never (nonswimmers)	793/833	1.00 (ref)	1805/1290	1.00 (ref)	674/528	1.00 (ref)
Low	267/216	0.93 (0.86–1.01)	411/106	0.86 (0.81–0.92)	100/85	1.06 (0.94–1.19)
Medium	97/76	0.94 (0.85–1.04)	172/50	0.87 (0.80–0.94)	57/27	0.94 (0.81–1.08)
High	79/77	1.00 (0.90–1.11)	136/69	0.94 (0.87–1.02)	41/55	1.23 (1.07–1.41)
*P* trend		0.981		0.001		0.407

a Co - controls; Ca - cases. Odds ratio (OR) and 95% confidence interval (CI) were calculated using mixed models with residential area as random effect and adjusted for age, sex, educational level, family history of cancer, smoking, energy intake, red and processed meat intake, body mass index, and physical activity. Never (nonswimmers) were those attending swimming pools <10 times in the lifetime. The cutoff points of Low, Medium and High pool attendance vary by cancer site and overall, summer and rest of the year (nonsummer). Overall pool attendance cut-offs (times/month) for breast cancer are 0 (never), >0–3.2 (low), 3.3–7.6 (medium), and >7.6 (high); for colorectal cancer are 0 (never), >0–2.1 (low), 2.2–7.6 (medium), and >7.7 (high); and for prostate cancer are 0 (never), >0–2.0 (low), 2.1–7.6 (medium), and >7.6 (high). Summer pool attendance cut-offs (times/month) for breast cancer are 0 (never), >0–8.6 (low), 8.7–17.3 (medium), and >17.3 (high); for colorectal cancer are 0 (never), >0–4.3 (low), 4.3–17.3 (medium), and >17.3 (high); and for prostate cancer are 0 (never), >0–4.3 (low), 4.3–13.0 (medium), and >13.0 (high). Nonsummer pool attendances (times/month) for breast cancer are 0 (never), >0–8.6 (low), 8.7–13.0 (medium), and >13.0 (high); for colorectal cancer are 0 (never), >0–8.6 (low), 8.7–13.0 (medium), and >13.0 (high); and for prostate cancer are 0 (never), >0–8.6 (low), 8.7–13.0 (medium), and >13.0 (high).

b Additionally adjusted for ever oral contraceptive use and menopausal status and treatment.

c Additionally adjusted for ever nonsteroidal anti-inflammatory drug consumption.

For breast and colorectal cancers, only pool attendance outside summer months was associated with a lower cancer risk (Table 2). The protective association of pool attendance with breast cancer was observed only in postmenopausal women (Table 3). Pool attendance appears to offer similar benefits for colorectal cancer prevention in both sexes, and stronger protective associations were observed among individuals reporting low physical activity (Table 3). On the other hand, the inverse association between breast cancer risk and high pool attendance was stronger among participants with high physical activity (Table 3).

**Table 3:** Stratified Analyses^a^

	Breast cancer^c^		Colorectal cancer^d^		Prostate cancer	
Lifetime swimming pool attendance	Co/Ca	OR (95% CI)	Co/Ca	OR (95% CI)	Co/Ca	OR (95% CI)
Physical Activity						
Low (<8 METs·h/week)						
Never (nonswimmers)	450/475	1.00 (ref)	917/719	1.00 (ref)	274/20	1.00 (ref)
Low	232/238	0.97 (0.92–1.03)	396/146	0.91 (0.86–0.95)	100/96	1.05 (0.97–1.15)
Medium	162/140	0.95 (0.88–1.01)	344/125	0.92 (0.87–0.96)	123/135	1.09 (1.01–1.19)
High	106/101	0.97 (0.90–1.05)	164/54	0.92 (0.86–0.98)	42/40	1.11 (0.99–1.24)
*P* trend		0.361		0.007		0.049
High (≥8 METs·h/week)						
Never (nonswimmers)	288/281	1.00 (ref)	736/446	1.00 (ref)	247/220	1.00 (ref)
Low	130/130	0.97 (0.90–1.05)	269/94	0.94 (0.89–1.00)	74/58	0.98 (0.89–1.08)
Medium	128/114	0.95 (0.88–1.02)	312/107	0.95 (0.90–1.00)	115/105	1.03 (0.95–1.12)
High	168/117	0.93 (0.86–0.99)	287/106	0.96 (0.91–1.02)	76/78	1.03 (0.95–1.13)
*P* trend		0.028		0.155		0.358
Interaction *p*-value[Table-fn t3fn5]		0.835		0.011		0.049
Menopausal Status						
Premenopause						
Never (nonswimmers)	162/193	1.00 (ref)				
Low	149/168	1.01 (0.93–1.01)				
Medium	93/110	1.02 (0.95–1.13)				
High	79/82	1.03 (0.93–1.12)				
*P* trend		0.682				
Postmenopause						
Never (nonswimmers)	574/560	1.00 (ref)				
Low	209/199	0.97 (0.91–1.03)				
Medium	195/143	0.91 (0.88–0.97)				
High	193/133	0.92 (0.87–0.98)				
*P* trend		0.003				
Interaction *p*-value[Table-fn t3fn5]		0.024				
Sex						
Females						
Never (nonswimmers)			768/436	1.00 (ref)		
Low			292/79	0.94 (0.89–1.00)		
Medium			366/89	0.93 (0.88–0.97)		
High			266/69	0.93 (0.88–0.99)		
*P* trend				0.014		
Males						
Never (nonswimmers)			885/729	1.00 (ref)		
Low			373/161	0.91 (0.86–0.95)		
Medium			290/143	0.95 (0.90–1.00)		
High			185/91	0.96 (0.90–1.02)		
*P* trend				0.280		
Interaction *p*-value[Table-fn t3fn5]				0.184		

a Association between breast, colorectal, and prostate cancer with average lifetime pool attendance (low, medium, high vs never) by physical activity, menopausal status, and sex. Co = Controls; Ca = Cases. Odds ratios (OR) and 95% confidence intervals (CI) calculated using mixed models with residential area as random effect adjusted for age, sex, educational level, family history of cancer, smoking, energy intake, red and processed meat intake, body mass index, and physical activity. The cutoff points of overall pool attendance (times/month) for breast cancer are 0 (never), >0–3.25 (low), 3.26–7.58 (medium), and >7.58 (high); for colorectal cancer are 0 (never), >0–2.16 (low), 2.17–7.58 (medium), and >7.58 (high); and for prostate cancer are 0 (never), >0–2.06 (low), 2.07–7.58 (medium), and >7.58 (high).

b Additionally adjusted for ever oral contraceptive use, menopausal status, and treatment.

c Additionally adjusted for ever nonsteroidal anti-inflammatory drug consumption.

d Calculated as the *p*-value of the multiplicative term between never/ever pool attendance and the stratifying variable.

When summer was separated from the rest of the year (Table 2), prostate cancer was associated with high frequency nonsummer swimming. Likewise, swimming was positively associated with prostate cancer among those with low physical activity (Table 3). No associations were found when stratifying by tumor grade (results are not shown).

## Discussion

Results from this large observational study, which included approximately 5000 cancer cases and 4000 controls, showed that participants who reported lifetime pool attendance showed approximately a 5% reduction in odds of developing breast and colorectal cancer compared to those who did not, while no protective effect was observed for prostate cancer. The dose–response analysis reveals that this protective association follows a linear pattern up to approximately 10 times per month (equivalent to 2–3 times per week), suggesting that regular moderate pool use may confer optimal benefits for both colorectal and breast cancers with a particularly strong protective association observed for postmenopausal breast cancer. The benefit appears to be limited to pool attendance all year round rather than during summer months. This is plausible given different behaviors expected in the summer, when pool attendance is more likely a recreational activity in outdoor pools, while swimming pool attendance during the rest of the year entails physical activity practice. In contrast, prostate cancer showed positive associations for high-frequency pool attendance during nonsummer periods.

Long-term exposure to DBP in tap water has been epidemiologically linked to increased risk of breast,^37^ colorectal,^38^ and prostate^39^ cancers. Although the IARC has classified several DBPs as possible human carcinogens, the underlying biological mechanisms remain inadequately characterized. Genotoxicity represents a primary mechanism of DBP carcinogenesis,^6^ and emerging research indicates that epigenetic modifications, particularly DNA methylation changes, may constitute an additional mechanism of carcinogenesis.^40^ Our group showed trihalomethane (THM) and haloacetic acid (HAA) uptake in biological samples from swimmers^11^ correlated with genotoxic responses,^12^ supporting the hypothesis that swimming in pools may represent an underexplored route of DBP exposure that deserves further investigation.

Swimming pools largely exceed DBP concentrations compared to public drinking water due to the constant organic matter input from swimmers and the continuous addition of disinfectant.^41^ In addition, the DBP composition differs from that in drinking water. N-DBPs (e.g., NDMA, halonitromethanes, haloacetonitriles) formed in swimming pools from urea and amino acids from bathers^42,43^ are more toxic than most THMs or HAAs. Although our large sample size, multiple study areas, and lifetime approach did not allow a comprehensive assessment of DBP exposure in biological and swimming pool samples, previous studies have extensively documented the presence of THMs and other DBPs in swimming pools in the study areas^30,44,45^ as well as the incorporation through inhalation and dermal absorption.^46^ Chlorine is the most widespread disinfectant used in swimming pools in Spain, particularly in the past, making THMs an adequate DBP marker. Studies comparing THM concentrations in water between indoor and outdoor pools report higher levels in outdoor facilities,^30^ that are frequently used in Spain during summer months. Elevated DBPs in outdoor pools may result from higher organic matter input or increased chlorination needs. However, despite higher water concentrations in outdoor pools, inhalation of THMs and volatile DBPs is likely greater in indoor pools due to accumulation in closed spaces.^30,47^

An experimental study conducted in one of the study areas found increased blood genotoxicity among volunteers after 40 min of swimming with stronger associations observed for brominated THMs.^2^ However, epidemiological studies evaluating the cancer risk associated with long-term swimming pool attendance are limited. A study in The Netherlands found a positive association between swimming history and melanoma,^48^ suggesting that carcinogenic agents in water, possibly DBPs, may play a role in melanoma etiology. A case-control study in Spain found that swimming in pools were associated with bladder cancer (OR = 1.57; 95% CI: 1.18–2.09), although exposure-response was not observed and collected information on lifetime pool attendance was limited.^49^ Additionally, an association between lymphocytic chronic leukemia and lifetime swimming pool attendance has been recently shown^50^ with participants who reported pool attendance having an OR = 2.38 (95%CI: 1.61–3.52) compared to those with no attendance.

Our findings suggest that regular swimming in pools reduces breast and colorectal cancer risk despite the potential carcinogenicity of DBPs. Physical activity is an established protective factor for breast and colorectal cancer,^19^ and this could explain the observed protective effect. Physical activity helps maintain a healthy weight, decreasing adiposity and chronic systemic inflammation—both known risk factors for cancer development. Additionally, exercise enhances immune surveillance by increasing the mobilization and activity of natural killer (NK) cells and cytotoxic T lymphocytes, which can identify and destroy emerging cancer cells before they establish tumors. Exercise also plays a crucial role in regulating hormones associated with cancer risk including insulin, estrogen, and insulin-like growth factor 1 (IGF-1). Lower levels of insulin and IGF-1 reduce cellular proliferation and mutation risk, while decreased estrogen exposure is particularly protective against breast cancer development. Furthermore, regular exercise is associated with improved DNA repair mechanisms and enhanced antioxidant capacity, both of which reduce the likelihood of genetic mutations that can initiate carcinogenesis.^51^

The current understanding of the long-term health effects of swimming pool use remains limited. This knowledge gap is particularly concerning for professional swimmers and frequent pool users who experience substantially higher DBP exposure due to the long time in the pool environment. Our dose–response analyses (Figure 2) suggest the possibility that high-frequency pool attendance may be associated with an elevated cancer risk. However, this finding must be interpreted with considerable caution, as the vast majority of participants in our study population reported low-to-moderate pool attendance frequencies with insufficient statistical power at higher exposure levels. To establish whether frequent pool use poses health risks, future research should specifically target participants with high-frequency exposure patterns, including professional swimmers and recreational athletes. Such studies would require larger sample sizes at high exposure levels and more detailed exposure assessment to adequately evaluate potential dose–response relationships.

Due to the case-control design and self-reported retrospective assessment, the main limitations of the present study are potential selection bias of controls and recall bias of exposure. However, similar baseline characteristics between cases and controls and the fact that participation was likely independent of exposure status suggest minimal selection bias, if any. Exposure assessment aimed to assess lifetime swimming pool habits, which was possible only through questionnaires. We used a proxy of exposure that aimed to capture both lifetime DBP exposure in swimming pools and physical activity during swimming by asking separately about indoor or outdoor pool attendance in summer and winter. Recall bias from self-reported retrospective assessment is expected to be nondifferential between cases and controls given that the link between swimming in pools and cancer was not obvious for participants. Although we tried to minimize exposure measurement error by excluding data from questionable interviews, associations probably have been attenuated toward the null. Furthermore, the small sample size among participants with swimming pool attendance exceeding 10 times per month requires cautious interpretation of findings and prevents us from properly evaluating the risks of high frequency pool use.

We acknowledge that multivariable adjustment, while essential for reducing confounding, cannot completely eliminate all potential sources of bias. Residual confounding may persist due to unmeasured variables or exposure measurement error in exposure assessment. Additionally, our models assume linear relationships and may not capture complex interactions between variables. Despite these limitations, our comprehensive adjustment strategy represents the current best practice for observational epidemiological studies and provides the most reliable estimates given the available data. This represents, to the best of our knowledge, the first epidemiological investigation of associations between lifetime swimming pool attendance and multiple prevalent cancer types. The study’s principal strengths include its large sample size, comprehensive assessment of lifetime pool exposure patterns, and wide range of adjusting covariates. The established long latency periods for breast, colorectal, and prostate cancers (10–20 years following carcinogenic exposure) support our lifetime exposure assessment approach, ensuring an adequate time for cancer development. These features complement existing smaller-scale and cross-sectional studies with more precise exposure quantification, collectively advancing our understanding of recreational water exposure and cancer risk.

## Supplementary Material



## Notes

Figure S1: Flowchart depicting the included and excluded study population; Table S1: Odds ratio (OR) and 95% confidence intervals (CI) of breast, colorectal, and prostate cancer associated with lifetime pool attendance vs nonattendance (i.e., <10 times in a lifetime) (PDF)

## Abbreviations

BMI: Body mass index
CI: confidence interval
DBPs: disinfection byproducts
ICD: International Classification of Diseases
GAMs: generalized additive models
METs: metabolic equivalents
THMs: trihalomethanes
OR: odds ratio

## References

[1] Richardson S., Thruston A., Caughran T., Chen P., Collette T., Schenck K., Lykins B., Rav-Acha C., Glezer V. (2000). Identification
of new drinking water disinfection by-products from ozone, chlorine
dioxide, chloramine, and chlorine. Water Air
Soil Pollut.

[2] Kogevinas M., Villanueva C. M., Font-Ribera L., Liviac D., Bustamante M., Espinoza F., Nieuwenhuijsen M. J., Espinosa A., Fernandez P., DeMarini D. M. (2010). Genotoxic effects in swimmers exposed to disinfection
by-products in indoor swimming pools. Environ.
Health Perspect.

[3] Richardson S. D., DeMarini D. M., Kogevinas M., Fernandez P., Marco E., Lourencetti C., Balleste C., Heederik D., Meliefste K., McKague A. B. (2010). What’s in the
pool? A comprehensive identification of disinfection by-products and
assessment of mutagenicity of chlorinated and brominated swimming
pool water. Environ. Health Perspect.

[4] Daiber E. J., DeMarini D. M., Ravuri S. A., Liberatore H. K., Cuthbertson A. A., Thompson-Klemish A., Byer J. D., Schmid J. E., Afifi M. Z., Blatchley E. R. (2016). Progressive Increase in Disinfection Byproducts
and Mutagenicity
from Source to Tap to Swimming Pool and Spa Water: Impact of Human
Inputs. Environ. Sci. Technol..

[5] Manasfi T., Coulomb B., Boudenne J. L. (2017). Occurrence, origin, and toxicity
of disinfection byproducts in chlorinated swimming pools: An overview. Int. J. Hyg Environ. Health.

[6] Richardson S. D., Plewa M. J., Wagner E. D., Schoeny R., Demarini D. M. (2007). Occurrence,
genotoxicity, and carcinogenicity of regulated and emerging disinfection
by-products in drinking water: a review and roadmap for research. Mutat. Res..

[7] Wilbourn J. D., Partensky C., Rice J. M. (1999). Agents that induce epithelial neoplasms
of the urinary bladder, renal cortex and thyroid follicular lining
in experimental animals and humans: summary of data from IARC monographs
volumes 1–69. IARC Sci. Publ.

[8] Working
Group on the Evaluation of Carcinogenic Risks to Human (IARC) (2013). Some chemicals present in industrial
and consumer products, food and drinking-water. IARC monographs on the evaluation of carcinogenic risks to humans.

[9] International
Agency for Research on Cancer (1999). Some chemicals that cause tumours of the kidney or urinary bladder
in rodents and some other substances. IARC Monographs
on the Evaluation of Carcinogenic Risk to Humans.

[10] Afifi M. Z., Blatchley E. R. (2015). Seasonal dynamics of water and air
chemistry in an
indoor chlorinated swimming pool. Water Res..

[11] Font-Ribera L., Kogevinas M., Schmalz C., Zwiener C., Marco E., Grimalt J. O., Liu J., Zhang X., Mitch W., Critelli R. (2016). Environmental
and personal determinants of
the uptake of disinfection by-products during swimming. Environ. Res..

[12] Font-Ribera L., Marco E., Grimalt J. O., Pastor S., Marcos R., Abramsson-Zetterberg L., Pedersen M., Grummt T., Junek R., Barreiro E. (2019). Exposure
to disinfection by-products in swimming
pools and biomarkers of genotoxicity and respiratory damage - The
PISCINA2 Study. Environ. Int..

[13] Vlaanderen J., van Veldhoven K., Font-Ribera L., Villanueva C. M., Chadeau-Hyam M., Portengen L., Grimalt J. O., Zwiener C., Heederik D., Zhang X. (2017). Acute changes in serum
immune markers due to swimming in a chlorinated pool. Environ. Int..

[14] Espin-Perez A., Font-Ribera L., van Veldhoven K., Krauskopf J., Portengen L., Chadeau-Hyam M., Vermeulen R., Grimalt J. O., Villanueva C. M., Vineis P. (2018). Blood
transcriptional and microRNA responses to short-term exposure to disinfection
by-products in a swimming pool. Environ. Int..

[15] van
Veldhoven K., Keski-Rahkonen P., Barupal D. K., Villanueva C. M., Font-Ribera L., Scalbert A., Bodinier B., Grimalt J. O., Zwiener C., Vlaanderen J. (2018). Effects of exposure
to water disinfection by-products in a swimming pool: A metabolome-wide
association study. Environ. Int..

[16] Liberatore H. K., Daiber E. J., Ravuri S. A., Schmid J. E., Richardson S. D., DeMarini D. M. (2022). Disinfection byproducts
in chlorinated or brominated
swimming pools and spas: Role of brominated DBPs and association with
mutagenicity. J. Environ. Sci. (China).

[17] Siegel R. L., Miller K. D., Wagle N. S., Jemal A. (2023). Cancer statistics,
2023. CA Cancer J. Clin.

[18] Sung H., Ferlay J., Siegel R. L., Laversanne M., Soerjomataram I., Jemal A., Bray F. (2021). Global Cancer
Statistics
2020: GLOBOCAN Estimates of Incidence and Mortality Worldwide for
36 Cancers in 185 Countries. CA Cancer J. Clin.

[19] Wild, C. P. ; Weiderpass, E. ; Stewart, B. W. , Eds. World Cancer Report: Cancer Research for Cancer Prevention; International Agency for Research on Cancer: Lyon, France, 2020. 39432694

[20] Sprague B. L., Trentham-Dietz A., Egan K. M., Titus-Ernstoff L., Hampton J. M., Newcomb P. A. (2008). Proportion of invasive breast cancer
attributable to risk factors modifiable after menopause. American journal of epidemiology.

[21] Barnes B. B., Steindorf K., Hein R., Flesch-Janys D., Chang-Claude J. (2011). Population
attributable risk of invasive postmenopausal
breast cancer and breast cancer subtypes for modifiable and non-modifiable
risk factors. Cancer Epidemiol.

[22] Keum N., Giovannucci E. (2019). Global burden
of colorectal cancer: emerging trends,
risk factors and prevention strategies. Nat.
Rev. Gastroenterol Hepatol.

[23] Gandaglia G., Leni R., Bray F., Fleshner N., Freedland S. J., Kibel A., Stattin P., Van Poppel H., La Vecchia C. (2021). Epidemiology and Prevention of Prostate
Cancer. Eur. Urol Oncol.

[24] Oja P., Memon A. R., Titze S., Jurakic D., Chen S. T., Shrestha N., Em S., Matolic T., Vasankari T., Heinonen A. (2024). Health
Benefits of Different Sports: a Systematic
Review and Meta-Analysis of Longitudinal and Intervention Studies
Including 2.6 Million Adult Participants. Sports
Med. Open.

[25] Gomez-Bruton A., Montero-Marin J., Gonzalez-Aguero A., Garcia-Campayo J., Moreno L. A., Casajus J. A., Vicente-Rodriguez G. (2016). The Effect
of Swimming During Childhood and Adolescence on Bone Mineral Density:
A Systematic Review and Meta-Analysis. Sports
Med..

[26] Lazar J. M., Khanna N., Chesler R., Salciccioli L. (2013). Swimming and
the heart. Int. J. Cardiol.

[27] Lee B. A., Oh D. J. (2015). Effect of regular
swimming exercise on the physical composition,
strength, and blood lipid of middle-aged women. J. Exerc Rehabil.

[28] Li J., Liu L., Cheng Y., Xie Q., Wu M., Chen X., Li Z., Chen H., Peng J., Shen A. (2022). Swimming attenuates
tumor growth in CT-26 tumor-bearing mice and suppresses angiogenesis
by mediating the HIF-1alpha/VEGFA pathway. Open
Life Sci..

[29] Villanueva C. M., Font-Ribera L. (2012). Health impact of disinfection by-products in swimming
pools. Ann. Ist Super Sanita.

[30] Font-Ribera L., Esplugues A., Ballester F., Martinez-Arguelles B., Tardon A., Freire C., Fernandez M. F., Carrasco G., Cases A., Sunyer J. (2010). [Trihalomethanes
in swimming pool water in four areas of Spain participating in the
INMA project]. Gac Sanit.

[31] Castano-Vinyals G., Aragones N., Perez-Gomez B., Martin V., Llorca J., Moreno V., Altzibar J. M., Ardanaz E., de Sanjose S., Jimenez-Moleon J. J. (2015). Population-based multicase-control study
in common tumors in Spain (MCC-Spain): rationale and study design. Gac Sanit.

[32] MCC-Spain study questionnaire version 18.0 [https://www.mccspain.org/wp-content/uploads/2016/07/Quest_MCCSpain.pdf]. Accessed 2025–10–17.

[33] Martin-Moreno J. M., Boyle P., Gorgojo L., Maisonneuve P., Fernandez-Rodriguez J. C., Salvini S., Willett W. C. (1993). Development
and validation of a food frequency questionnaire in Spain. Int. J. Epidemiol.

[34] Epstein J. I., Egevad L., Amin M. B., Delahunt B., Srigley J. R., Humphrey P. A., Grading C. (2016). The 2014 International
Society of
Urological Pathology (ISUP) Consensus Conference on Gleason Grading
of Prostatic Carcinoma: Definition of Grading Patterns and Proposal
for a New Grading System. Am. J. Surg Pathol.

[35] Hurwitz L. M., Agalliu I., Albanes D., Barry K. H., Berndt S. I., Cai Q., Chen C., Cheng I., Genkinger J. M., Giles G. G. (2021). Recommended Definitions of Aggressive Prostate
Cancer for Etiologic Epidemiologic Research. J. Natl. Cancer Inst.

[36] Hayati
Rezvan P., Lee K. J., Simpson J. A. (2015). The rise of multiple
imputation: a review of the reporting and implementation of the method
in medical research. BMC Med. Res. Methodol.

[37] Font-Ribera L., Gracia-Lavedan E., Aragones N., Perez-Gomez B., Pollan M., Amiano P., Jimenez-Zabala A., Castano-Vinyals G., Roca-Barcelo A., Ardanaz E. (2018). Long-term
exposure to trihalomethanes in drinking water and breast cancer in
the Spanish multicase-control study on cancer (MCC-SPAIN). Environ. Int..

[38] Villanueva C. M., Gracia-Lavedan E., Bosetti C., Righi E., Molina A. J., Martin V., Boldo E., Aragones N., Perez-Gomez B., Pollan M. (2017). Colorectal Cancer and
Long-Term Exposure to Trihalomethanes
in Drinking Water: A Multicenter Case-Control Study in Spain and Italy. Environ. Health Perspect.

[39] Donat-Vargas C., Kogevinas M., Castano-Vinyals G., Perez-Gomez B., Llorca J., Vanaclocha-Espi M., Fernandez-Tardon G., Costas L., Aragones N., Gomez-Acebo I. (2023). Long-Term Exposure to Nitrate and Trihalomethanes in Drinking Water
and Prostate Cancer: A Multicase-Control Study in Spain (MCC-Spain). Environ. Health Perspect.

[40] Salas L. A., Bustamante M., Gonzalez J. R., Gracia-Lavedan E., Moreno V., Kogevinas M., Villanueva C. M. (2015). DNA methylation
levels and long-term trihalomethane exposure in drinking water: an
epigenome-wide association study. Epigenetics.

[41] Carter R. A. A., Allard S., Croue J. P., Joll C. A. (2019). Occurrence of disinfection
by-products in swimming pools and the estimated resulting cytotoxicity. Sci. Total Environ..

[42] Jurado-Sanchez B., Ballesteros E., Gallego M. (2010). Screening of N-nitrosamines in tap
and swimming pool waters using fast gas chromatography. J. Sep Sci..

[43] Walse S. S., Mitch W. A. (2008). Nitrosamine carcinogens
also swim in chlorinated pools. Environ. Sci.
Technol..

[44] Santa
Marina L., Ibarluzea J., Basterrechea M., Goni F., Ulibarrena E., Artieda J., Orruno I. (2009). [Indoor air
and bathing water pollution in indoor swimming pools in Guipuzcoa
(Spain)]. Gac Sanit.

[45] Abilleira E., Goni-Irigoyen F., Aurrekoetxea J. J., Cortes M. A., Ayerdi M., Ibarluzea J. (2023). Swimming pool
water disinfection by-products profiles
and association patterns. Heliyon.

[46] Caro J., Gallego M. (2007). Assessment of exposure
of workers and swimmers to trihalomethanes
in an indoor swimming pool. Environ. Sci. Technol..

[47] Erdinger L., Kuhn K. P., Kirsch F., Feldhues R., Frobel T., Nohynek B., Gabrio T. (2004). Pathways of
trihalomethane uptake
in swimming pools. Int. J. Hyg Environ. Health.

[48] Nelemans P. J., Rampen F. H., Groenendal H., Kiemeney L. A., Ruiter D. J., Verbeek A. L. (1994). Swimming and the risk of cutaneous melanoma. Melanoma Res..

[49] Villanueva C. M., Cantor K. P., Grimalt J. O., Malats N., Silverman D., Tardon A., Garcia-Closas R., Serra C., Carrato A., Castano-Vinyals G. (2006). Bladder cancer and exposure to water disinfection
by-products through ingestion, bathing, showering, and swimming in
pools. American journal of epidemiology.

[50] Donat-Vargas C., Kogevinas M., Benavente Y., Costas L., Campo E., Castano-Vinyals G., Fernandez-Tardon G., Llorca J., Gomez-Acebo I., Aragones N. (2024). Lifetime exposure to brominated trihalomethanes
in drinking water and swimming pool attendance are associated with
chronic lymphocytic leukemia: a Multicase-Control Study in Spain (MCC-Spain). J. Expo Sci. Environ. Epidemiol.

[51] Spanoudaki M., Giaginis C., Karafyllaki D., Papadopoulos K., Solovos E., Antasouras G., Sfikas G., Papadopoulos A. N., Papadopoulou S. K. (2023). Exercise
as a Promising Agent against Cancer: Evaluating
Its Anti-Cancer Molecular Mechanisms. Cancers
(Basel).

