# Supplementary material for: Lifetime Swimming Pool Attendance and Cancer Risk: Findings from the Multicase-Control Study in Spain (MCC-Spain)
Source: Environ Sci Technol. 2025 Nov 3;59(45):24257–67. doi: 10.1021/acs.est.5c06488 (PMC12631987; doi:10.1021/acs.est.5c06488)

Supporting Information

Lifetime swimming pool attendance and cancer risk: Findings from the Multicase–Control Study in Spain (MCC-Spain)

Carolina Donat-Vargas[1,2,3,4], Miquel Vallbona-Vistós[1,5], Gemma Castaño-Vinyals[1,3,5,6], Víctor Moreno[3,7,8], Nuria Aragonés[3,9], Elena Boldo[3,10,11], Antonio José Molina de la Torre[3,12], Inés Gómez-Acebo[3,13], Marcela Guevara[14,15], Ana Jiménez Zabala[16], Pilar Amiano[3,16], Ana Molina-Barceló[17], Guillermo Fernández-Tardón[3,18], Maria Dolores Chirlaque[3,19,20], Marina Pollán[3,11], Manolis Kogevinas[1,3,5,6], Cristina M Villanueva[1,3,5,6].

1) ISGlobal, Doctor Aiguader 88, 08003-Barcelona, Spain.
2) Polyphenol Research Group, Department of Nutrition, Food Sciences and Gastronomy, School of Pharmacy and Food Sciences, University of Barcelona, Campus Diagonal, Av. de Joan XXIII, 27-31, Distrito de Les Corts, 08028 Barcelona, Spain; INSA-UB, Nutrition and Food Safety Research Institute, Carrer de Prat de la Riba, 171, 08921 Santa Coloma de Gramanet, Spain.
3) CIBER de Epidemiología y Salud Pública (CIBERESP), Instituto de Salud Carlos III, Av. Monforte de Lemos, 3-5. Pabellón 11. 28029-Madrid, Spain.
4) Unit of Cardiovascular and Nutritional Epidemiology, Institute of Environmental Medicine, Karolinska Institutet, Nobels väg 13, 171 65 Solna, Stockholm, Sweden.
5) Universitat Pompeu Fabra (UPF), Doctor Aiguader 88, 08003-Barcelona, Spain
6) IMIM (Hospital del Mar Medical Research Institute), Doctor Aiguader 88, 08003-Barcelona, Spain.
7) University of Barcelona, Feixa Llarga s/n, 08907-L'Hospitalet de Llobregat, Barcelona, Spain
8) Catalan Institute of Oncology, Bellvitge Biomedical Research Institute (IDIBELL), Gran Via km 2.7, 08907-L' Hospitalet de Llobregat, Barcelona, Spain.
9) Cancer Surveillance Unit, Public Health Division, Department of Health of Madrid, Madrid, Spain.
10) Cancer Epidemiology Research Group, Oncology and Hematology Area, Instituto de Investigación Sanitaria (IIS) Puerta De Hierro, C/Joaquín Rodrigo 2, 28222-Madrid, Spain.
11) Cancer and Environmental Epidemiology Unit, National Centre for Epidemiology, Carlos III Institute of Health, Monforte de Lemos 5, 28029-Madrid, Spain.
12) Research Group in Gene–Environment–Health Interactions (GIIGAS), University of Leon, Altos de Nava s/n, 24071León, Spain.
13) Preventive Medicine Group, University of Cantabria, Santander, Spain. IDIVAL-Valdecilla HealthResearch Institute, Avenida Cardenal Herrera Oria s/n, 39011-Santander, Spain
14) Navarra Public Health Institute, Leyre 15, 31003-Pamplona, Spain.
15) Navarra Institute for Health Research (IdiSNA), Francisco Bergamín 2 bis, 31003-Pamplona, Spain.
16) Public Health Division of Gipuzkoa, Biogipuzkoa Research Institute, Av. Navarra 4, 20013-San Sebastian, Spain.
17) Cancer and Public Health Area, Foundation for the Promotion of Health and Biomedical Research-Public Health Research (FISABIO), Avda. de Catalunya 21, 46020-Valencia, Spain.

18) Health Research Institute of Asturias (ISPA), Av. Hospital Universitario s/n, 33011-Oviedo, Asturias, Spain.
19) Department of Epidemiology, Murcia Health Council, IMIB-Arrixaca, Ronda de Levante 11, 300008-Murcia, Spain.
20) Department of Health and Social Sciences, Universidad de Murcia, Av. Teniente Flomesta 5, 30003-Murcia, Spain

Number of pages: 4

Number of figures: 1

Number of tables: 1

Table S1. Odds ratio (OR) and 95% confidence intervals (CI) of breast, colorectal and prostate cancer associated with lifetime pool attendance vs. non-attendance (i.e. <10 times in a lifetime).

**Lifetime swimming pool attendance**	**Co/Ca**	**OR (95%CI)[1]**	**OR (95%CI)[2]**	**OR (95%CI)[3]**
Breast cancer				
No users	738/756	1.00 (ref)	1.00 (ref)	1.00 (ref)
Users	1161/968	0.93 (0.89, 0.96)	0.94 (0.90, 0.97)	0.94 (0.91, 0.97)
Colorectal cancer				
No users	1653/1165	1.00 (ref)	1.00 (ref)	1.00 (ref)
Users	2274/946	0.95 (0.92, 0.97)	0.95 (0.92, 0.97)	0.95 (0.93, 0.98)
Prostate cancer				
No users	611/490	1.00 (ref)	1.00 (ref)	1.00 (ref)
Users	877/616	0.97 (0.94, 1.02)	0.98 (0.94, 1.02)	0.98 (0.94, 1.02)

Co=Controls; Ca=Cases

Odds ratios (OR) and 95% confidence intervals (CI) were calculated using mixed models with residential area as random effect.

[1] adjusted for age, sex and educational level

[2] further adjusted for family history of cancer, smoking, energy intake, red and processed meat intake, body mass index

[3] further adjusted for physical activity

Breast cancer is additionally adjusted for ever oral contraceptive use and menopausal status and treatment; and colorectal cancer for nonsteroidal anti-inflammatory drug consumption.

Figure S1. Flow chart depicting the included and excluded study population

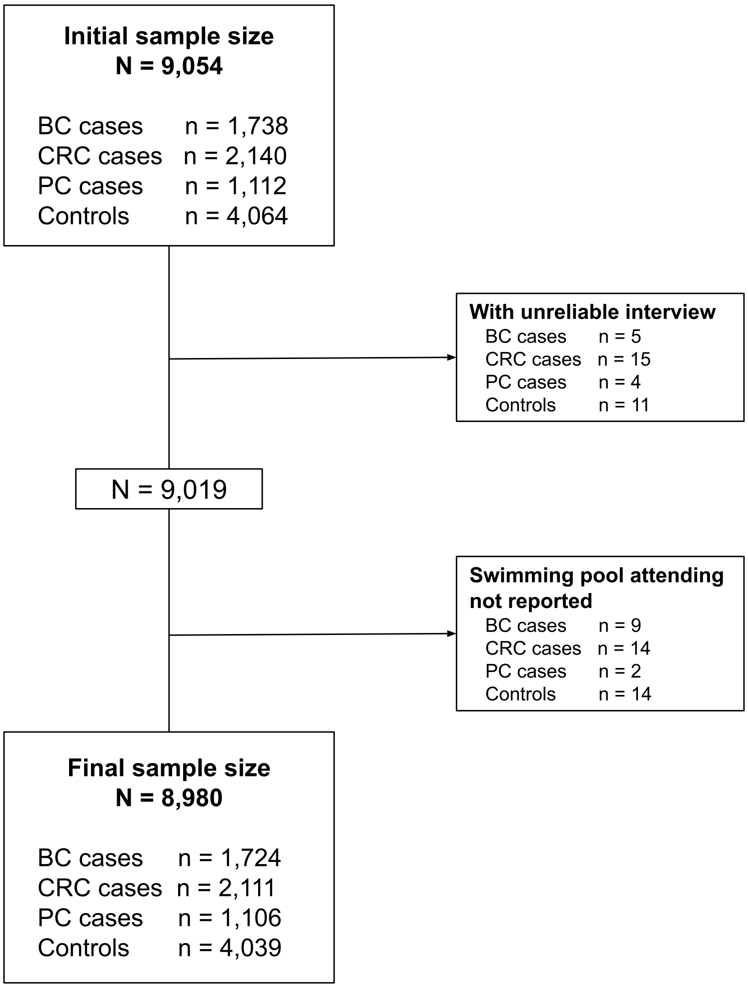

Supplement: Supplementary file 1 [file es5c06488_si_001.pdf]
